# The effect of MD1 on potassium and L-type calcium current of cardiomyocytes from high-fat diet mice

**DOI:** 10.1080/19336950.2020.1772628

**Published:** 2020-06-03

**Authors:** Wei Shuai, Bin Kong, Hui Fu, Xiaobo Jiang, He Huang

**Affiliations:** a Department of Cardiology, Renmin Hospital of Wuhan University, Wuhan, Hubei, China; b Cardiovascular Research Institute of Wuhan University, Wuhan, Hubei, China; c Hubei Key Laboratory of Cardiology, Wuhan, Hubei, China

**Keywords:** Myeloid differentiation protein 1, high-fat diet, potassium current, l-type calcium current, ventricular arrhythmias

## Abstract

Myeloid differentiation protein 1 (MD1) is exerted an anti-arrhythmic effect in obese mice. Therefore, we sought to clarify whether MD1 can alter the electrophysiological remodeling of cardiac myocytes from obese mice by regulating voltage-gated potassium current and calcium current. MD1 knock-out (KO) and wild type (WT) mice were given a high-fat diet (HFD) for 20 weeks, starting at the age of 6 weeks. The potential electrophysiological mechanisms were estimated by whole-cell patch-clamp and molecular analysis. After 20-week HFD feeding, action potential duration (APD) from left ventricular myocytes of MD1-KO mice revealed APD_20_, APD_50_, and APD_90_ were profoundly enlarged. Furthermore, HFD mice showed a decrease in the fast transient outward potassium currents (I_to,f_), slowly inactivating potassium current (I_K, slow_), and inward rectifier potassium current (I_K1_). Besides, HFD-fed mice showed that the current density of I_CaL_ was significantly lower, and the haft inactivation voltage was markedly shifted right. These HFD induced above adverse effects were further exacerbated in KO mice. The mRNA expression of potassium ion channels (Kv4.2, Kv4.3, Kv2.1, Kv1.5, and Kir2.1) and calcium ion channel (Cav1.2) was markedly decreased in MD1-KO HFD-fed mice. MD1 deletion led to down-regulated potassium currents and slowed inactivation of L-type calcium channel in an obese mice model.

## Introduction

Obesity can lead to metabolic syndrome and result in cardiac dysfunction in humans and animal models [1]. Obesity can also markedly increase the risk of ventricular arrhythmias (VA) and sudden cardiac death (SCD) [2,3]. However, the key molecules involved in VA caused by HFD-induced obesity are yet to be determined.

Myeloid differentiation protein 1 (MD1) is a type of secreted glycoprotein, and it can form a complex with radioprotective protein 105 (RP105) [4]. The MD1-RP105 complex can directly interact with the myeloid differentiation protein 2 (MD2)-Toll-like receptor 4 (TLR4) complex by a lateral binding, acting as a negative physiological regulator of the TLR4 signaling pathway [5]. Our previous studies found that MD1 expression was downregulated in heart failure patients [6,7], and loss of MD1 could worsen structural and electrical remodeling under pressure overload and ischemia/reperfusion injury conditions via increased the activation of the TLR4 signaling pathway [6,8]. Interestingly, we also found that MD1 deletion had no significant effect on the cardiac structure in wild-type (WT) mice under physiological conditions [6–11]. Moreover, downregulated MD1 does not activate the TLR4 signaling pathway in both in vitro and in vivo experiments [7,9]. Our current study also observed that MD1 is downregulated in the obese mouse heart, and MD1 protected cardiac function against high-fat diet (HFD)-induced pathological remodeling [9,11]. Furthermore, MD1 deletion could also significantly prolong action potential duration (APD) and increase susceptibility to VA through enhanced activation of the TLR4/MyD88/CaMKII signaling pathway in the *ex vivo* Langendorff-perfused hearts of HFD-induced obese mice [10]. However, the potential electrophysiological mechanisms of MD1 in HFD-fed induced VA have not been determined. The current study demonstrated the mechanistic relationship between MD1 and obesity-related VA through investigated potassium channels and L-type calcium channels (LTCCs).

## Materials and methods

An expanded Methods section describing all procedures and protocols can be found in the ***Supplemental Methods*** section.

### Experimental animals

**Figure 1.**
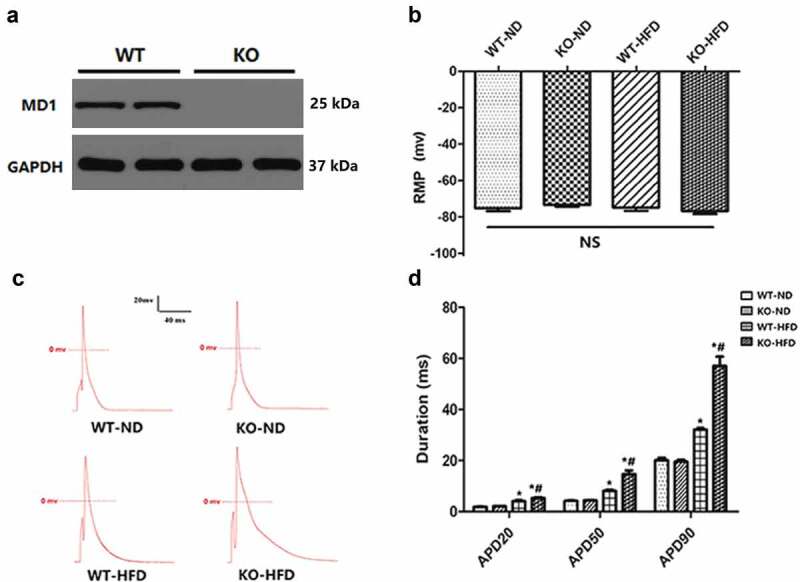
**MD1 Deletion alters action potential durations in ventricular cardiomyocytes isolated from WT and KO mouse hearts after 20 weeks of ND or HFD feeding**. (a) Representative western blots of MD1 expression in LV tissues from WT and MD1-KO mice (n = 6). (b) Changes of resting membrane potential (RMP) (n = 8 cardiomyocytes from n = 4 mice each group). (c, d) Representative action potential figures and statistical analysis of the 20%, 50%, and 90% action potential durations (n = 8 cardiomyocytes from n = 4 mice each group). Data are expressed as mean ± SEM. * *p* < 0.05 vs. WT-ND group, # *p* < 0.05 vs. WT-HFD group.

All experiments involving animals have conformed to the guidelines established by the Guide for the Care and Use of Laboratory Animals published by the US National Institutes of Health (The 8^th^ Edition, NRC 2011) and approved by the Animal Care and Use Committee of Renmin Hospital at Wuhan University. MD1 knock-out (MD1-KO) mice were generated as described by previous studies [6,7,10,11]. Male MD1-KO mice were purchased from the Japan RIKEN BioResource Centre Mouse (BRC) (B6.129P2-MD-1<tm1Kmiy>). All of the mice were housed in a surrounding with controlled light cycles (12 h light/12 h dark), temperature, and humidity. The MD1-KO mice were confirmed by Western blot (Figure 1(a)).

### Statistical analysis

The data analyzes were performed with SPSS24 or GraphPad Prism software. Continuous variables are expressed as means ± SEM. Statistical differences between 2 groups were determined by Student’s t-test. Statistical comparisons among multiple groups were performed with one-way analysis of variance (ANOVA), followed by Tukey’s post hoc test. Significance was assumed when *p* < 0.05.

## Results

### The effect of MD1 deletion on APD in the ventricular myocytes of the WT and KO mice after 20 weeks ND or HFD feeding

HFD feeding markedly increased the body weight, glycemic and lipid levels in WT-HFD mice vs. WT-ND mice (P < 0.05, Table 1). These HFD-fed induced obesity-caused metabolic disorders were further worsened in KO-HFD mice vs. WT-HFD mice (P < 0.05, Table 1). Our previous finding showed that loss of MD1 significantly prolonged APD in ex vivo Langendorff-perfused hearts of HFD-fed mice [10]. To clarify the above mechanisms, we first recorded the single-cell action potential (AP) and resting membrane potential (RMP) in ventricular myocytes. Figure 1(b) showed that the RMP in 4 groups were comparable. Figure 1(c) showed representative AP recorded in ventricular myocytes derived from the four groups of mice. As depicted in Figure 1(d), APD_20_, APD_50_, and APD_90_ were dramatically prolonged in WT-HFD vs. WT-ND mice (*p* < 0.05) and KO-HFD vs. KO-ND mice (*p* < 0.05). The prolongation in KO-HFD mice was significantly attenuated in the WT-HFD mice. These results indicated that MD1 deficiency could markedly prolong the APD of ventricular myocytes in the setting of HFD feeding.

**Table 1.** The characteristic of HFD-fed induced obese mice model.

	WT-ND	KO-ND	WT-HFD	KO-HFD
BW, g	30.49 ± 0.29	31.29 ± 0.31	35.7 ± 0.3*	46.31 ± 0.55*#
Glucose, mmol/l	6.11 ± 0.23	6.21 ± 0.12	7.5 ± 0.15*	9.71 ± 0.19*#
TC, mmol/L	2.0 ± 0.12	1.97 ± 0.05	3.8 ± 0.19*	5.18 ± 0.2*#
TG, mmol/L	0.69 ± 0.03	0.73 ± 0.03	1.45 ± 0.08*	2 ± 0.06*#
LDL-c, mmol/L	0.83 ± 0.06	0.83 ± 0.02	1.7 ± 0.05*	1.95 ± 0.06*#

N = 8 for each group. Data are presented as mean ± SEM. BW: body weight; TC, total cholesterol; TG, triglyceride; LDL-c: low-density lipoprotein cholesterol; HFD, high-fat diet; * *p* < 0.05 vs. WT-ND group, # *p* < 0.05 vs. WT-HFD group.

### The current density of outward potassium current in the ventricular myocytes of the WT and KO mice after 20 weeks of ND or HFD feeding

**Figure 2.**
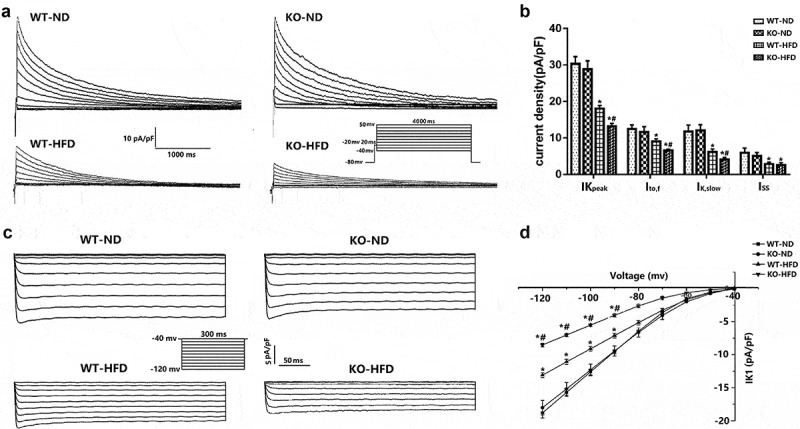
**Altered outward potassium currents and inward rectifier potassium currents in ventricular cardiomyocytes isolated from WT and KO mouse hearts after 20 weeks of ND or HFD feeding**. a) Typical examples of voltage-dependent outward potassium current tracings. b) Graph of the current density of outward potassium currents (n = 8 cardiomyocytes from n = 3 mice each group). c) Typical examples of the inward rectifier potassium current recorded in a whole-cell configuration. d) Graph of the current density of inward rectifier potassium current (n = 8 cardiomyocytes from n = 3 mice each group). I_Kpeak_, peak outward potassium current; I_to, f_, fast transient outward potassium currents; I_K, slow_, slowly inactivating potassium current; I_ss_, non-inactivating current component; I_k1_, inward rectified potassium currents. Data are expressed as mean ± SEM. * *p* < 0.05 vs. WT-ND group, # *p* < 0.05 vs. WT-HFD group.

Because decreased repolarizing potassium current density contributed to APD prolongation, we further characterized the major repolarizing outward potassium current, fast transient outward potassium currents (I_to, f_), slowly inactivating potassium current (I_K, slow_), and non-inactivating steady-state outward potassium current (I_ss_), in isolated ventricular cardiomyocytes derived from 4 groups of mice. After HFD feeding, the current densities of I_to, f,_ and I_K, slow_ were significantly decreased in KO mice as compared with their relative ND control (Figure 2(a, b)). However, there was no significant change in I_ss_ in the two groups. There were no significant differences in I_to, f_, I_K, slow_, and I_ss_ between WT-ND and KO-ND mice. Collectively, these results indicate that MD1 deficiency could decrease the current densities of I_to, f,_ and I_K, slow_, which could be responsible for the prolonged APD in ventricular cardiomyocytes after HFD feeding.

### The current density of inward rectifier potassium current (I_K1_) in the ventricular myocytes of the WT and KO mice after 20 weeks of ND or HFD feeding

I_K1_ was also found to play an important role in determining cardiac membrane potential and the terminal phase of membrane repolarization (represented by APD_90_) [12]. Figure 1(d) showed that APD_90_ was dramatically prolonged in the cardiomyocytes isolated from KO-HFD mice. Therefore, we further characterized the current densities of I_K1_ in 4 groups of mice. In the present study, we found that the current density of I_K1_ was markedly decreased at voltages ranging from −80 mV to −120 mV in KO-HFD mice compared with WT-HFD mice (Figure 2(c, d)). There was no difference in the current density of I_K1_ between the WT-ND and KO-ND mice (Figure 2(c, d)). In brief, these results indicate that the MD1 deficiency could decrease I_K1_, which could be responsible for the prolonged APD in ventricular cardiomyocytes after HFD feeding.

### The current density of I_CaL_ in the ventricular myocytes of the WT and KO mice after 20 weeks of ND or HFD feeding

**Figure 3.**
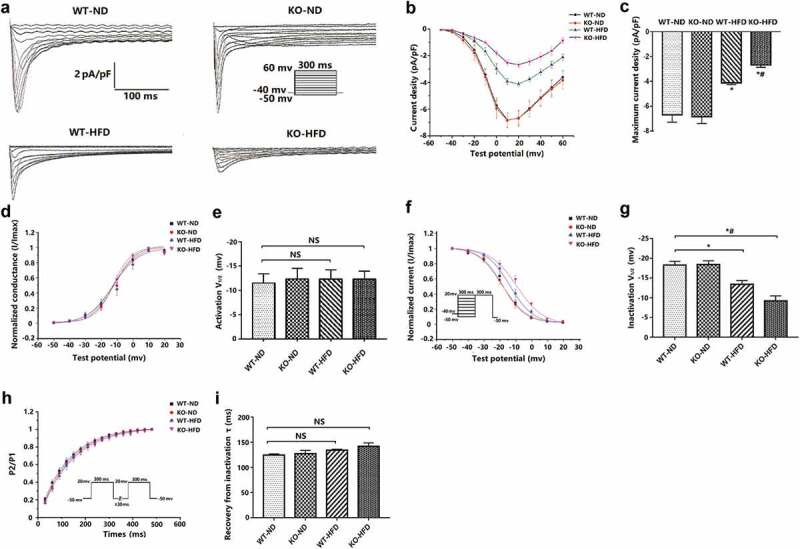
**Altered L-type calcium current density and gating properties in the ventricular myocytes of the WT and KO mice after 20 weeks of ND or HFD feeding**. a) Typical examples of L-type calcium current tracings. b) Current density-voltage (i–v) correlation for L-type calcium current. c) Maximum L-type calcium current density (n = 8 cardiomyocytes from n = 3 mice each group). d) The steady-state L-type calcium channel activation curves. Curves were fit to the Boltzmann function (n = 8 cardiomyocytes from n = 3 mice each group). e) the haft activation voltage of the L-type calcium channel (V1/2). f) the steady-state L-type calcium channel inactivation curves, which were determined with the double-pulse protocol (Hold potential was −40 mv. A 300 ms pre-pulse of potentials between −50 and +20 mV in 10 mV steps, followed by a fixed 300 ms test pulse of 20 mV). Curves were fit to the Boltzmann function (n = 8 cardiomyocytes from n = 3 mice each group). g) the haft inactivation voltage of the L-type calcium channel (V1/2). h) the recovery curves following L-type calcium channel inactivation, which were determined with the double-pulse protocol (Hold potential was −50 mv. A 300 ms inactivating pulse (+ 20 mv), were followed at intervals from 30 to 480 ms in 30-ms increments by an identical test pulse). Curves were fit to a mono-exponential function (n = 8 cardiomyocytes from n = 3 mice each group). i) the recovery time constant (τ) for the L-type calcium channel. Data are expressed as mean ± SEM. * *p* < 0.05 vs. WT-ND group, # *p* < 0.05 vs. WT-HFD group.

Prolonged cardiomyocyte APD results from either an increased inward ionic flow, a decreased outward current, or a combination of both. We focused on the LTCCs because of their primary role in calcium influx. Our results showed that the maximum current density (Figure 3(c)) was of significantly lower amplitude in the WT-HFD mice than the WT-ND mice (−4.11 ± 0.14 pA/pF vs. −6.69 ± 0.6 pA/pF, *p* < 0.05), and the I_CaL_ amplitude was significantly lower in KO-HFD mice than the WT- HFD mice (−2.67 ± 0.2 pA/pF vs. −4.11 ± 0.14 pA/pF, *p* < 0.05). No significant differences were found between WT-ND and KO-ND mice (−6.69 ± 0.6 pA/pF vs. −6.85 ± 0.56 pA/pF, *p* > 0.05).

### Kinetic characteristics of LTCCs in the ventricular myocytes of the WT and KO mice after 20 weeks of ND or HFD feeding

The above results showed that the peak current density of inward current I_CaL_ was decreased after HFD feeding, but the APD of cardiomyocytes was prolonged (the prolongation of APD was related to the increase of inward current). We speculate that it may be due to the change of ion channel dynamics of LTCCs. Therefore, we further detected the kinetic characteristics of LTCCs (Table 2). The inactivation curve of KO-HFD mice was markedly right-shifted relative to the WT-HFD group (Figure 3(f), V1/2: −9.2 ± 1.27 mv vs. −13.42 ± 0.94 mv, *p* < 0.05). No significant differences were found between WT-ND and KO-ND mice (Figure 3(f), V1/2: −18.29 ± 0.93 mv vs. −18.42 ± 0.95 mv, *p* > 0.05). Additionally, the half activation voltage (Figure 3(e)) and recovery time constant (Figure 3(i)) were not significant in the four groups (*p* > 0.05).

**Table 2.** Effect of loss MD1 based on obese model on ICaL channel kinetics.

	WT-ND	KO-ND	WT-HFD	KO-HFD
Activation V_1/2_ (mv)	−11.53 ± 1.89	−12.36 ± 2.2	−12.29 ± 1.99	−12.31 ± 1.66
Inactivation V_1/2_ (mv)	−18.29 ± 0.93	−18.49 ± 0.95	−13.48 ± 0.94*	−9.2 ± 1.27*#
Recovery from inactivation τ (ms)	124.74 ± 2.19	127.4 ± 6.4	130.81 ± 1.61	141.84 ± 6.91

Values presented are mean± SEM (n = 8 cells/group), * *p* < 0.05 vs. WT-ND group, # *p* < 0.05 vs. WT-HFD group.

### MD1 deficiency affected the mRNA expression of cardiac ion channels after HFD feeding

**Figure 4.**
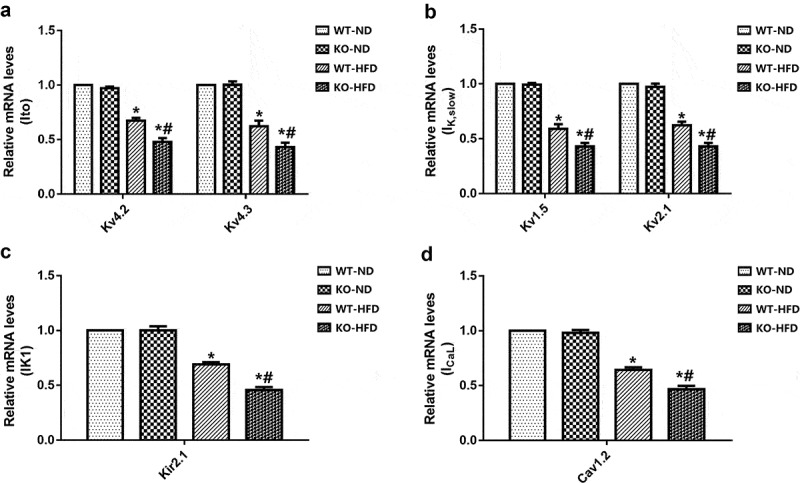
**Expression of channels in WT and KO mouse hearts after 20 weeks of ND or HFD feeding**. (a-c) The mRNA levels of the potassium channel subunits of transient outward potassium current I_to,f_ (Kv4.2, Kv4.3), delayed rectifier potassium current I_K, slow_ (Kv1.5 and Kv2.1), and inwardly rectified potassium current I_K1_ (Kir2.1). (d) The mRNA levels of the calcium channel subunit of L-type calcium current I_CaL_ (Cav1.2). N = 4 mice in each group. Data are expressed as mean ± SEM. * *p* < 0.05 vs. WT-ND group, # *p* < 0.05 vs. WT-HFD group.

Finally, we assessed the molecular mechanisms by profiling repolarized ion channel gene expression in the WT and KO mice after 20 weeks of ND or HFD feeding. The mRNA expression of I_to,f_ (Kv4.2, Kv4.3), I_K, slow_ (Kv1.5 and Kv2.1), and I_K1_ (Kir2.1) in KO-HFD mice were significantly lower than that of WT-HFD mice. Moreover, the mRNA expression of I_CaL_ (Cav1.2) was significantly decreased in KO-HFD mice compared with WT-HFD mice. There was no difference in the mRNA expression of all of the above ion channels between the WT-ND and KO-ND mice (Figure 4). Taken together, loss of MD1 was found to suppress the mRNA expression of potassium ion channels and LTCCs in HFD-fed mice.

## Discussion

The TLR4-mediated signal transduction pathway plays an essential role in cardiac structural and electrical remodeling caused by obesity [13,14]. Our previous studies illuminated that loss of MD1, a negative physiological regulator of the TLR4 signaling pathway, could worsen cardiac structural and electrical remodeling in a pathological condition, such as hypertrophic cardiomyopathy and ischemia/reperfusion injury [6,8]. Our current study found that MD1 is downregulated in the hearts of obese mice [9,11], and loss of MD1 markedly prolonged APD and increased susceptibility to VA via enhanced activation of the TLR4/MyD88/CaMKII signaling pathway in HFD-fed mice [10]. The present study further demonstrated the potential electrophysiological remodeling mechanisms between MD1 and obesity-related VA. The main findings of the study are as follows: 1) Loss of MD1 had no significant effect on the electrophysiological remodeling of cardiac myocytes from ND-fed mice. 2) Deletion of MD1 prolonged ventricular myocyte APD in the HFD-fed mice. 3) The Deletion of MD1 decreased the current density of outward potassium current and I_K1_ in HFD-fed mice. 4) KO-HFD mice showed the decreased current density of I_CaL_ and slowed inactivation of LTCCs. 5) Loss of MD1 suppressed the expression of potassium ion channels and LTCCs in HFD-fed mice. These findings hinted that MD1 might play a vital role in the electrophysiological remodeling of cardiomyocytes from HFD-fed mice.

Obesity-related cardiac remodeling is the important pathological manifestation of sudden cardiac death (SCD) [15]. In earlier work, our group found that HFD could induce pathological cardiac remodeling and increase susceptibility to VA [9]. Moreover, excessive APD prolongation is a hallmark of the abnormally altered electrophysiology or adverse electrical remodeling [16,17]. Our previous study found that MD1 deficiency decreased the protein expressions of Kv4.2, Kv4.3, Kv1.5, Kv2.1, and Cav1.2 channels by the enhanced activation of the TLR4/MyD88/CaMKII signaling pathway [10]. To further clarify the effect of MD1 on obesity-related electrophysiological characteristics, we have isolated mouse ventricular myocytes for cell electrophysiology experiments. In the present research, single-cell recordings showed that APD_20_, APD_50_, and APD_90_ were markedly prolonged in WT-HFD mice compared with WT-ND mice. Our result is in agreement with a previous work by Bai and coworkers in myocytes isolated from HFD-fed rat hearts [18]. In their experiments, APD_90_ was markedly elongated by HFD. A recent study also suggested that the activation of cardiac TLR4 by lipopolysaccharides (LPS) significantly increases the AP duration [13]. Moreover, we also found that after HFD feeding, the APD of KO mice was markedly prolonged compared with WT mice. This was similar to our previous findings, which demonstrated that MD1 deficiency could worsen prolonged APD *in vivo* experiments [10]. Therefore, we believed that MD1 deletion might alter the electrophysiological properties of the cardiomyocytes of HFD-fed mice.

As we all know, prolonged AP duration results from either an increased inward ionic flow, a decreased outward current, or a combination of both. Several studies have shown that reducing the proteins of outward potassium channels would induce a significant reduction of outward potassium current density, consequently prolonging the AP [19,20]. In the present experiment, we found that the current densities and mRNA levels of I_to, f_ and I_K, slow_ were significantly decreased in HFD mice. Moreover, the decrease was further exacerbated in KO mice compared with WT mice. This is similar to our previous findings, which found that the protein levels of I_to, f_ (Kv4.2, Kv4.3) and I_K, slow_ (Kv2.1 and Kv1.5) was markedly decreased in KO-HFD mice [10]. Similar results were also reported by Grandinetti et al., who suggested that decreases in Ito would delay repolarization and prolong APD, making dysregulation of Ito, a plausible contributor to long QT syndrome (LQTS) in obese patients [21]. Moreover, a recent study also indicates that HFD-fed mouse hearts showed decreased protein levels of Kv4.2 [22]. Finally, using DIO mice, Huang et al. also demonstrated impaired ventricular repolarization, which was associated with reduced mRNA levels of Kv1.5 channel subunits [23]. In consequence, these data suggested that loss of MD1 led to the exacerbation in the electrophysiological remodeling of cardiomyocytes after HFD feeding through downregulation of the current densities and mRNA levels of outward potassium channels.

I_K1_ plays an essential role in cardiac excitability and arrhythmogenesis and is a promising target for new anti-arrhythmic approaches [24]. A recent study showed that I_K1_ plays a crucial role in modulating the RMP and the final repolarization phase of AP in cardiomyocytes [25]. In the current study, we found that the current densities and mRNA levels of I_K1_ were significantly decreased in HFD mice, and the decrease was further exacerbated in HFD-fed MD-KO mice. These observations are consistent with studies by Bai et al. showing that I_K1_ current densities and Kir2.1 protein expression were significantly decreased in HFD rats, which results in the APD_90_ prolongation. In contrast, another study demonstrated significantly increased mRNA levels of Kir2.1 channel subunit and I_K1_ current density in an HFD obese rat model [26]. The specific reasons behind these conflicting results are not clear. They could involve species differences and differences in experimental conditions. Further experiments are needed to explain these differences and define the specific mechanisms of compartmentalized regulation of I_K1_ in normal and diseased hearts.

Our results also showed that I_CaL_ current densities and Cav1.2 mRNA expression were decreased after HFD feeding, which was minimal in MD1-KO HFD-fed mice. This is similar to our earlier work, which showed that HFD could reduce the protein expression of Cav1.2 in MD1-KO mice compared to WT mice [10]. Supporting these data, a recent report by Lin et al. has shown that the calcium channel protein levels and I_CaL_ current density were decreased in obese rats [27]. Moreover, several studies have demonstrated that calcium channel protein levels and I_CaL_ current density were reduced in the obese animal model [22,28,29]. I_CaL_, which contributes to phase 2 depolarization of AP, was reduced, resulting in the APD shortening. However, the APD of our findings is extended. In this study, we found that the current densities of outward potassium and mRNA levels of potassium ion channels in the HFD-fed heart were downregulated, resulting in a decreased outward current, which may exert a stronger effect on APD than increased inward I_CaL_ current. The kinetic characteristics of LTCCs showed that the inactivation curve of KO-HFD mice was markedly right-shifted relative to the WT-HFD, which could lead to the existence of continuous inward current. Recent research has reported that although the peak current of I_CaL_ was decreasing, the APD prolonged due to constant inward current caused by defective calcium inactivation [27]. Therefore, we believe that reduced outward potassium current and interfered I_CaL_ inactivation played significant roles in the prolongation of cardiac AP in the progression of HFD-induced obesity hearts. However, all of the above findings are inconsistent with studies by Ricci et al., who reported that there was no significant difference in the current densities of I_to, f_, I_K, slow,_ and I_CaL_ in ventricular myocytes from the HFD-fed group compared to the ND group [30]. There are several possible reasons for these differences. The first is that the fat energy supply ratio of the HFD selected by the researcher was different from that of our study (33% vs. 60%), and the duration of HFD feeding in their study was 14 weeks, while that was 20 weeks in our study, all of which may lead to different degrees of obesity and related metabolic disorders. The other is that different experimental animals were used in the studies. The object of their research was the rat, while our study used mice. Therefore, in future research, we should combine different preclinical models of obesity to further explore the obesity-induced cardiac electrophysiological remodeling.

## Study limitations

The current study had several limitations. The study revealed that loss of MD1 could increase electrophysiological remodeling of cardiomyocytes from HFD-fed mice by downregulating potassium currents and slowing the inactivation of I_CaL_; however, there were no clear and definitive mechanistic insights into how the effects of the loss of MD1 regulate the function and expression of ion channels. Future studies are required to elucidate further the detailed mechanism by which MD1 regulates ion channels via the TLR4/CaMKII signaling pathway.

## Conclusions

In summary, the present research indicated that MD1 deficiency led to downregulated potassium currents and slowed inactivation of I_CaL_ in an obese mice model, which may be the mechanism of electrophysiological remodeling for increased vulnerability to HFD-fed induce VA.

## Supplementary Material

Supplemental Material
