# Supplementary material for: The effect of MD1 on potassium and L-type calcium current of cardiomyocytes from high-fat diet mice
Source: Channels (Austin). 2020 Jun 3;14(1):181–9. doi: 10.1080/19336950.2020.1772628 (PMC7515570; doi:10.1080/19336950.2020.1772628)
Supplement: Supplemental Material [file KCHL_A_1772628_SM6932.docx]

**Supplementary materials**

**Supplementary methods**

**1.1 Experimental animals**

All experiments involving animals have conformed to the guidelines established by the Guide for the Care and Use of Laboratory Animals published by the US National Institutes of Health (The 8^th^ Edition, NRC 2011) and approved by the Animal Care and Use Committee of Renmin Hospital at Wuhan University. MD1 knock-out (MD1-KO) mice were generated as described by previous studies [1-4]. Male MD1-KO mice were purchased from the Japan RIKEN BioResource Centre Mouse (BRC) (B6.129P2-MD-1<tm1Kmiy>). All of the mice were housed in a surrounding with controlled light cycles (12 h light/12 h dark), temperature, and humidity. The diﬀerent groups of mice were respectively fed with an HFD (60% kcal from fat) and a normal diet (ND) containing 10% of kcal from the fat beginning at the age of 6 weeks. HFD and age-matched ND mice were weighed weekly. The levels of fast glucose, total cholesterol (TC), triglyceride (TG), and low-density lipoprotein cholesterol (LDL-c) were evaluated when they reached 26 weeks of age [2].

**1.2 Isolation of cardiomyocytes**

Cardiomyocytes were isolated using previously described methods [1]. In brief, mice were anesthetized by intraperitoneal injection of sodium pentobarbital (60 mg/kg). The hearts were excised rapidly and connected to a Langendroff apparatus with a constant flow at a rate of 2-3 mL/min for retrograde perfusion via the aorta, at 37℃ for 5 min in Ca2^+^-free Tyrode’s solution containing with (in mmol/L): NaCl 130; KCl 5.4; MgCl_2_ 1; Na_2_HPO_4_ 0.3; HEPES 10; glucose 10; PH adjusted to 7.35 with NaOH. The hearts were further digested with the same solution containing 0.3 mg/ml collagenase type II (Sigma, Co.US), 0.1% bovine serum albumin, and 30 μM of CaCl_2_ for 15-20 min. At the end of the perfusion, the left ventricular (LV) free wall was dissected from the heart and placed in cold KB solution (mM: taurine 10; glutamic acid 70; creatine 0.5; succinic acid 5; dextrose 10; KH_2_PO_4_ 10; KCl 20; HEPES 10; EGTA 0.2; PH adjusted to 7.35 with KOH). Cardiomyocytes were separated by pipetting, and calcium was reintroduced to suspend cells by gradual increases to a final concentration of 1 mM. The cardiomyocyte suspension was stored in the KB solution at 4°C before the electrophysiology test.

**1.3 Cellular electrophysiology recording**

Whole-cell patch-clamp was performed using the EPC-9 amplifier (List Instruments, Germany), and data were analyzed with the Pulse-fit software interface (Version 8.31, HEKA Co. Germany). The resistances of the pipettes ranged from 3 to 6 MΩ when filled with pipette solution. Series resistance (Rs) was between 4-10 MΩ, and compensation was applied to reduce Rs by 80-90%. Current signals were filtered at 3 kHz by an 8-pole Bessel filter, digitized at a sampling rate of 1 kHz, stored on the computer running Pulse software, which was used for the generation of pulses. Data analysis was used Clamp-fit 10.7 and Origin 9.0.

For action potential (AP) recordings, we measured induced AP with the whole-cell current-clamp mode, and the myocytes were continuously superfused with the normal Tyrode’s solution. The pipette solution contained the following (mM): K-aspartate 110; KCl 20; NaCl 8; MgCl_2_ 1; CaCl_2_ 1; Mg-ATP 4; EGTA 0.1; HEPES 10; PH 7.2 with KOH. The following AP parameters were analyzed: APD at 20%, 50%, and 90% of repolarization (APD_20_, APD_50_, and APD_90_, respectively).

The potassium current recordings were similar to the published methods [5]. The external solution contained: NaCl 140 mM, KCl 4 mM, MgCl_2_ 1 mM, NaH_2_PO_4_ 0.33 mM, CaCl_2_ 1.8 mM, HEPES 10 mM, glucose 10 mM, and pH adjusted to 7.4 with NaOH. The pipette solution contained (in mM): KCl 140, MgCl_2_ 1, HEPES 10, EGTA 10, Mg-ATP 5, and pH adjusted to 7.2 with KOH. A brief (20 ms) voltage step to -20 mV was used to activate and inactivate sodium channels. Voltage-dependent potassium currents were elicited with voltage steps between -50 mV and +60 mV in 10-mv increments, for 4 s with a 10-s interval. The decay phases of the outward potassium currents evoked during the 4.0 s depolarizing voltage step to +50 mV were fit by a double exponential function of the form:

Y(t)=A1 exp(-t/τ1) + A2 exp(-t/τ2) +A_ss_

where t is time, τ1 and τ2 are the decay time constants, A1 and A2 are the amplitudes of fast transient outward potassium currents (I_to, f_), and slowly inactivates the potassium current (I_K, slow_), respectively, and A_ss_ is the amplitude of the non-inactivating steady-state outward potassium current (I_ss_).

To measure the inward rectifier potassium current (I_K1_), myocytes were held at a membrane potential of -40 mV (to inactivate sodium and calcium channels) and voltage steps from -120 to -40 mV in 10-mV increments were applied for 300 ms with an inter-pulse interval of 1 s. I_K1_ current amplitude (pA) was calculated after subtracting the background currents in the presence of 100 μΜ of BaCl2. Current density (pA/pF) was calculated from the current amplitude (pA) divided by the membrane capacitance (pF).

L-type calcium current (I_CaL_) was measured using the whole-cell voltage-clamp mode. The external solution contained (in mM): choline chloride 100, NaCl 35, NaH_2_PO_4_ 0.33 MgCl_2_ 1, KCl 5.4, CaCl_2_ 1.8, HEPES 10, glucose 10, BaCl_2_ 0.1, 4-aminopyridine 5, and Ph 7.4 with NaOH. The pipette solution contained (in mM): CsCl 120, EGTA 10, CaCl_2_ 1, MgCl_2_ 5, Na_2_-ATP 5, HEPES 10, and pH 7.2 with CsOH. The interval between pulses was 5 s. The whole-cell patch-clamp of I_CaL_ analyses included 4 parameters for each experimental group: (1) current-voltage relationship curves, (2) steady-state activation curves, and (3) steady-state inactivation curves, and these data were also fitted to the Boltzmann distribution to obtain the half activation(V1/2)) and (4) recovery curves from inactivation.

**1.4 Reverse-transcription quantitative polymerase chain reaction (RT-qPCR)**

Total RNA was extracted from LV using TRIzol reagent (cat. no. 15596-026; Thermo Fisher Scientific, Inc.) and was reverse transcribed into cDNA using the PrimeScript RT reagent kit (cat. no. RR047A; Takara Biotechnology Co., Ltd.). RT-qPCR was subsequently performed using a 20-μl reaction system containing cDNA, forward and reverse primers and SYBR^®^ Premix Ex Taq (cat. no. RR420A; Takara Biotechnology Co., Ltd.). Glyceraldehyde 3-phosphate dehydrogenase (GAPDH) was used as an internal reference gene. The sequences of the primers used for RT-qPCR are presented in ***Supplementary Table S1.***

**1.5 Western blotting analysis**

Total protein was extracted and separated on sodium dodecyl sulfate polyacrylamide gel electrophoresis (SDS-PAGE, AS1086, ASPEN). Then, proteins were transferred onto polyvinylidene difluoride (PVDF) membranes (EMD Millipore, Billerica, MA). The membranes were incubated overnight at 4℃ with the primary antibody of MD1 (LS-B6457-50, LSBIO). Finally, secondary antibodies of GAPDH (AB37168, ABCAM) were incubated with the membranes for 30 minutes at room temperature. Protein bands were detected using chemiluminescence (cat. no. NCI 5079; Thermo Fisher Scientific, Inc.).

**Supplementary Table**

Table S1 Mouse Primers for RT-PCR

| Gene | Forward Primers | Reverse Primers |
| --- | --- | --- |
| Kv4.2 | CTACGCAGAGAAGGGCTCTTC | AAGACTCCGCTCAGTGAGCAG |
| Kv4.3 | TCTTCTCTTCTCCCTTACCATGG | AGCAGATGGAGCCAAATATCTTC |
| Kv2.1 | AACACACTGCCTGAGCTACAGAG | AAGTACTCCATGGTGAACCACG |
| Kv1.5 | ATGAAGGCTTCATCAAGGAAGAG | CAGAGTCTCCAAGCAGAAGGTG |
| Kir2.1 | CAGTGTGAGAACCAACCGCTAC | ATCTTTCTTCACAAAGCGGCTC |
| Cav1.2 | TCCTGGAGGTTCCAACTATGG | TGACGGTAGAGATGGTTGCG |
| GAPDH | TGAAGGGTGGAGCCAAAAG | AGTCTTCTGGGTGGCAGTGAT |

**Reference**

[1] Peng JY, Liu Y, Xiong XJ, Huang CX, Mei Y, Wang ZQ, et al. Loss of MD1 exacerbates pressure overload-induced left ventricular structural and electrical remodelling. Sci Rep. 2017;7.

[2] Shuai W, Kong B, Fu H, Shen CJ, Jiang XB, Huang H. MD1 Deficiency Promotes Inflammatory Atrial Remodelling Induced by High-Fat Diets. Can J Cardiol. 2019;35:208-16.

[3] Xiong X, Liu Y, Mei Y, Peng J, Wang Z, Kong B, et al. Novel Protective Role of Myeloid Differentiation 1 in Pathological Cardiac Remodelling. Sci Rep. 2017;7:41857.

[4] Shuai W, Kong B, Fu H, Shen C, Huang H. Loss of MD1 increases vulnerability to ventricular arrhythmia in diet-induced obesity mice via enhanced activation of the TLR4/MyD88/CaMKII signaling pathway. Nutr Metab Cardiovasc Dis 2019;29:991-998.

[5] Morrow JP, Katchman A, Son NH, Trent CM, Khan R, Shiomi T, et al. Mice With Cardiac Overexpression of Peroxisome Proliferator-Activated Receptor gamma Have Impaired Repolarization and Spontaneous Fatal Ventricular Arrhythmias. Circulation. 2011;124:2812-U123.
